# Cultural variation in factors associated with sudden infant death during sleep

**DOI:** 10.1186/s12887-021-02894-8

**Published:** 2021-10-09

**Authors:** Young Mee Ahn, Kyung-moo Yang, Hong Il Ha, Jung Ae Cho

**Affiliations:** 1 Department of Nursing, Inha University, 100 Inha-ro, Michuhol-gu, Incheon, 22212 Republic of Korea; 2 Division of Forensic Medicine, National Forensic Service, 10, Ipchun-ro, Wonju-si, Gangwon-do Republic of Korea 26460; 3 Division of Forensic Medicine, National Forensic Service Seoul Institute, 139, Jiyang-ro, Yangcheon-gu, Seoul, Republic of Korea 08036; 4 Department of Nursing, Inha University, 100 Inha-ro, Michuhol-gu, Incheon, 22212 Republic of Korea

**Keywords:** Sudden unexpected death in infancy, Infant sleep practices, Culture

## Abstract

**Background:**

Despite the significant reduction decades ago in sudden unexpected death in infancy (SUDI), decline of rates has slowed and stalled in some countries, including the USA. This led to an appreciation of ethnic variations in SUDI rates and the need to increase cultural sensitivity regarding sleep practices and circumstantial factors of SUDI. The study explored SUDI-related factors, in journal articles from two geo-cultural regions (Asian and Western countries), particularly for factors related to infant sleep practices.

**Methods:**

A systematic review was conducted to identify SUDI-related factors in articles from PubMed, Scopus, and the Korean Citation Index from January 1992 to April 2019. From each article, SUDI-related factors were retrieved and categorized through the identification, aggregation, and categorization of factors into the areas of the triple risk model (TRM) of SUDI by their meanings and commonality. Significant trends in the frequency of factors were analyzed across time and between the two geo-cultural regions (Asian and Western countries) of article.

**Results:**

From a review of 218 articles (38 Asian and 180 Western articles), 84 SUDI-related factors were identified: 39 factors for TRM 1, 44 factors for TRM 2, and one factor for TRM 3. Four of the top-ranked 10 factors were found in both cultural zones: sleep position, male sex, bed-sharing, and genetics. Both cultural zones identified sleep position (44.0%), bed-sharing (22.0%), and rooming-in (16.5%) as the three most important sleep-related factors for SUDI. Variations between the cultural zones were observed in the place of SUDI occurrence, overheating, swaddling or bedding standards, and smoking.

**Conclusions:**

Regardless of the urgent need to identify SUDI-related factors in low-SUDI societies, Asian cultures showed a significant lack of articles on SUDI. Several sociocultural issues were recognized such as the meaning of bed-sharing and rooming-in, along with residential styles and traditional health beliefs on sleep-related SUDI factors. Particularly little attention towards smoking was found in Asian articles in terms of frequency, suggesting the need to enhance SUDI reduction strategies by incorporating gender-sensitive smoking cessation interventions. This review of SUDI factors requests child health professionals to be alert to sociocultural variations in sleep practices and SUDI factors.

## Background

Sudden infant death syndrome (SIDS) refers to infant deaths during sleep where the cause is unknown despite a thorough exploration of the infant’s clinical history, an investigation of the death scene, and an autopsy [1]. As a similar-sounding, but distinct term, sudden unexpected death in infancy (SUDI) refers to the death of an infant in which the cause of death cannot be identified as a known medical condition or another readily identifiable factor [2]. The term SUDI is generally used at the time of presentation; therefore, in some cases, a cause of death is found, whereas other cases may remain categorized as unexplained SUDI [2].

The diagnosis of either SIDS or SUDI depends on the quality of the social infrastructure, including the responsibility and capacity of related professionals to investigate cases of infants who suddenly die for unknown reasons. Some nations have reported fairly accurate estimations of the incidence of SIDS/SUDI, including 3600 annual SUDI cases in the US [2] and 200 annual SIDS cases in the UK [3]. In Korea, the National Forensic Service reported rates of 0.2 [4] or 0.22–0.36 per 1000 births for SUDI, including SIDS, based on autopsies [5]. However, reliable and accurate diagnoses require meticulous investigations using advanced technology, which is not possible in cases with no investigation of the death scene or a circumstantial review including a physical assessment of the infant and sleep practices of the infant and family. Therefore, it is difficult to make accurate statistical estimations of the incidence of SIDS or SUDI and to compare those rates across societies.

Numerous efforts have been made to explore the mechanisms responsible for unexplained infant deaths and to identify underlying factors associated with SUDI. The first significant steps to explore SUDI were made in Western societies such as the US [6], UK [7, 8], and Australia [9] where incidents of infant death during sleep without any clear explanation have been an important public concern. The well-known triple risk model (TRM) developed by Filiano and Kinney [10] explicates otherwise unexplained infant death during sleep in terms of a combination of three triggering risk factors: vulnerability of the infant, exogenous stressors, and the critical developmental period. In particular, the role of exogenous stressors has received attention for the development of behavioral and cognitive interventions by health care professionals as tangible steps that can be taken to reduce SUDI [11, 12]. As an example, the “safe sleep” (previously “back to sleep”) campaign has been successfully adopted in Western societies to minimize infant death from accidental suffocation during sleep, along with emphasizing other relevant factors from the three areas of TRM during recent decades [13].

However, after the noticeable decrease in SUDI in response to the back to sleep campaign in the late 1990s, the SUDI rates have stabilized in some societies. The stagnation of SUDI rates suggested that ethnicity may play an independent role in SUDI rates. Ethnic variation in SUDI was identified even in the very early era of SIDS research 5 decades ago, when the SUDI rate per 1000 births was found to be 0.51 in Asians, 1.32 in Whites, and 5.93 in American Indians [14]. In 4 recent years of SUDI data from the US, Asian/Pacific Islander infants had a lower SUDI rate than infants of other ethnic origins [2]. Data from England and Wales from 2006 to 2012 showed a nearly fivefold variation in SUDI incidence across ethnic groups, with the lowest incidence found in infants with Indian, Bangladeshi, or Pakistani ethnicity [15]. This variation was not attributable to the well-known SUDI-related factors in the TRM, such as preterm birth, maternal age, or low socioeconomic status (SES). Based on the assumption that immigrants from Asian/Pacific or East-Asian areas may live in relatively less affluent conditions in Western societies, these reports suggest that material deficiency may not translate to a low-quality infant and child caregiving environment and may not qualify as an inherent risk factor for SUDI. Instead, this finding implies that it is necessary to explore the infant and child caregiving practices and environment of low-SUDI societies to develop SUDI reduction strategies.

Sleep practices are cultural phenomena, which are transmitted through long-term processes in a given society, shaped by familial resources social structures, and environmental factors. Our previous article addressed the cultural issues accounting for SIDS in respect to infant caring practices (ICP) and residential structures across societies and cultures [16]. The article specifically dealt with cultural variations in sleep practices, with significant implications for SIDS reduction strategies between Western and Asian countries. Its findings support the need for special attention to understand the factors associated with sleep-related infant death in the context of sleep practices as part of ICP in various cultures. However, some Asian countries have yet to establish a statutory system and professional atmosphere regarding SUDI, whereas countries such as the UK or US have relatively strong legislative systems pertaining to SUDI that have sparked more in-depth research on SUDI. Therefore, it may be appropriate at this point in time to investigate SUDI-related factors that have been reported over time, to assess how they differ between Asian cultures and other societies, and then to analyze the current state of the art regarding SUDI-related factors in publications. Through such an analysis, it may be possible to derive suggestions on important points to consider. This study was conducted to identify the SUDI-related factors reported in journal articles since the first guideline for SIDS was introduced by the American Academy of Pediatrics (AAP) and to analyze the trends therein by time and between two geo-cultural regions (Asian and Western countries), with a particular focus on factors related to sleep practices as part of ICP.

## Methods

### Search conditions and article database construction

We conducted a systematic review to identify and compare SUDI-related factors from articles according to geo-spatial region and times. PubMed and Scopus were used as databases for international articles. For Korean articles (with English-language abstracts and tables), the Korean Citation Index (KCI, https://www.kci.go.kr/kciportal/main.kci), Research Information Sharing Service (RISS, http://www.riss.kr/index.do), and DBpia (http://www.dbpia.co.kr) were used.

For article retrieval, we applied the semantic approach to identify SUDI-related factors meaningfully addressed in articles. In order to consider the possibility of database overlap between PubMed and Scopus and to emphasize factors likely to be identified as causing SUDI after an investigation, we used the following key words for the search query with modifications in the search conditions: from PubMed, “SIDS,” “sleep death,” “sudden infant death syndrome,” and “risk factor” in the title or abstract in combination with “AND” and “OR” operators, and from Scopus, “sudden infant death syndrome,” “sleep death,” “SIDS,” and “cause,” all in a combined form. The period for the search was from January 1992, when AAP published the first guideline on reducing SIDS [17] to April 2019. Two meta-analyses on SIDS were also reviewed to identify any other new and relevant articles.

**Fig. 1:**
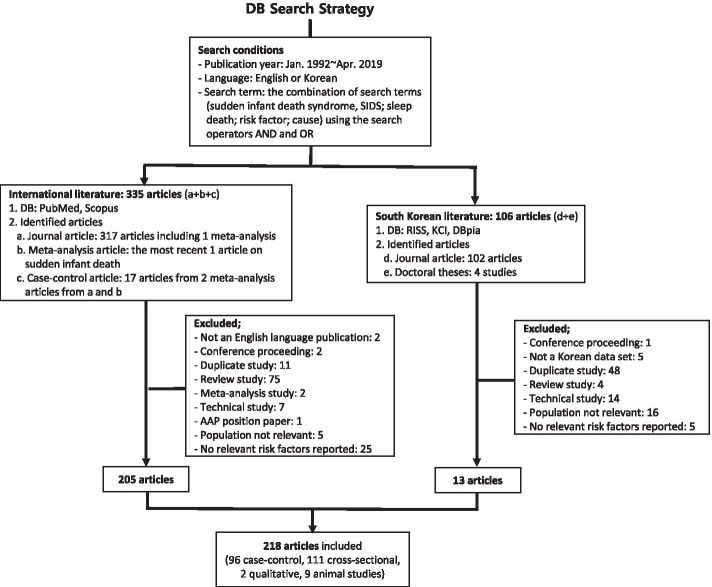
Database search strategy flowchart

Each article was screened for full-text availability and originality of articles. Short comments, conference proceedings, and position papers from professional organizations such as AAP were excluded as well as article studies by the same authors with analogous themes and contents. Since this process required a qualitative assessment of articles while maintaining a robust framework and clear content for a systematic review of SUDI articles, the screening process involved a critical and thorough analysis of each article by two authors (YMA, JAC), the results of which were verified among all authors, rather than an automated computer-based elimination process. Fig. 1 presents the inclusion conditions of retrieving articles from databases and the exclusion conditions among those retrieved articles.

### Factor retrieval and categorization process

The factor retrieval process involved three steps: identification, aggregation, and categorization of factors into scopes and TRM types. First, we identified risk or related factors as the actual words in which they were presented based on the main themes, key words, or primary variables from each article. We did not specifically search the factors according to a certain framework, such as PICO (participant, intervention, comparison and outcomes), or evaluate statistical significance, since study design or hypothesis testing on SUDI was not our primary subject of inquiry. However, factors that were not main concepts, but showed statistical significance or meaningful interpretations in previous articles, were included.

Second, if some factors were presented using different wording across articles, but still shared the same or a very similar meaning, we aggregated them as a single factor based on their meanings and commonality regarding SUDI. The depth and coverage of aggregated factors varied since each article used its own terms for key concepts depending on its specific purpose and the content. For example, one study could present a factor encompassing a broad range of phenomena (e.g., ‘infection’), while another could present a very specific factor (e.g., ‘weekday’). An example of factor aggregation is the factor of ‘event place,’ which comprised several specific locations (car seat, sofa, couch, *ondol*, and swing) from various articles. Another example of aggregation is that in some cases, a single underlying factor (such as sleep position) could be presented as either a risk factor (i.e., prone position) or as a protective factor (e.g., supine position) for sleep-related infant death. We included both risky and protective factors within a single factor (such as sleep position) in such cases. Table 6 also summarizes some examples of aggregation of sleep-related factors from the actual words contained in the articles.

Third, the factors were combined into broad scopes if their nature and impact shared a substantial core related to SUDI. The scopes were then classified according to the three factors in the TRM of SUDI. One example of factor categorization was the scope of clinical factors (infection, apnea, vaccination, etc.), which was placed into the category of exogenous stressors of TRM 2. Table 1 shows an example of the factor retrieval and categorization process.

**Table 1:** Factor Retrieval and Categorization Process^a^

Steps	Process	Example 1		Example 2
Identification of the actual wording	Extraction of key words, themes, or concepts regarding SUDI-related factors from each article	Brainstem gliosis, hypoplasia of the hypoglossus nucleus, hypodevelopment of the arcuate nucleus, etc.		Car seat, sofa, couch, *ondol*, swing, etc.
Aggregation of factors	Semantic analysis and merging synonyms by meanings and commonality regarding SUDI	Brainstem problems		Event place
Categorization of factors	Combination of the factors into scopes and TRM according to their nature and impact on SUDI	Scopes	Abnormality, dysfunctionality	Circumstantial factors
		TRM	TRM 1: Vulnerability of the infant	TRM 2: Exogenous stressors

^a^Additional examples in Table 6

We also collected information on the country of the study, the publication year of the article, and characteristics of the subjects and study design in order to analyze trends in SUDI-related factors in articles over time and between the two cultural regions of interest (Asian and Western countries).

In order to maintain reliability and validity throughout the process of retrieving and classifying factors, systematic decision-making rules were established to guide back-and-forth transitions across the three steps while identifying the hundreds of actual terms from articles and placing them into aggregated factors, relevant scopes, and TRMs in a consistent manner. The factor retrieval and categorization processes were independently performed twice by two authors (YMA, JAC). Any discrepancies within or between reviewers were discussed among authors and the decision-making rules were revalidated to obtain the final agreement.

A database was generated containing factors, scopes and TRMs with the bibliographic information of articles, including study countries. To analyze trends in SUDI factors between the two cultural regions, we initially grouped the countries of the articles into four regions (Asia, America, Europe, and Oceania) based on their geographical location. This classification was then merged into a division between two geo-spatial cultural zones (Asian and Western). Some articles dealt with multiethnic groups or ethnic variations in SUDI-related phenomena. Nonetheless, we consistently used the country of the study to allocate articles into two geo-cultural regions, rather than interpreting and selecting the culture-specific findings from each indicated article, because any such SUDI-related factors would be clearly identifiable throughout the articles.

Data were analyzed using IBM SPSS Statistics 26 (IBM Corp., Armonk, NY, USA) for descriptive statistics of variations in SUDI-related factors in articles across time, countries, and two geo-cultural regions, and the factors presented graphically using Tableau for quantitative, visually engaging comparisons of significant trends. We also used the general term “SUDI” to describe and discuss the findings of this study to reflect the inclusion of the maximum possible breadth of relevant studies, including those on SIDS, SUDI in sleep, or any analogous cases.

## Results

Using the above search conditions, 335 international articles (317 articles: 94 in PubMed, 223 in Scopus, two meta-analyses, and 17 articles from the two meta-analyses) and 106 Korean articles, including 4 dissertations, were identified. Finally, a total of 218 articles was included, of which 96 were case-control studies, 111 were cross-sectional descriptive studies, two were qualitative studies, and nine were animal studies. From 218 articles, we identified 84 factors and 10 scopes for the 3 TRMs of SUDI as shown in Table 2. TRM 1 (vulnerability of the infant) included 39 factors in the following 5 scopes: abnormality (dysfunctionality), birth problems, genetic/familial factors, general issues, and the prenatal environment. For TRM 2 (exogenous stressors), 4 scopes and 44 factors were recognized: Ecological factors, circumstantial factors (feeding, sleeping, or surroundings), clinical issues, and SES. Finally, TRM 3 (critical period of SIDS) was considered to be a single factor, which was addressed by 69 (31.7%) of the 218 articles. In Table 2, the parentheses for specific factors refer to the frequency of articles in which they were mentioned, while the parentheses for TRM category and scopes indicate the number of factors in each group.

**Table 2:** Categorization of SUDI Factors by the Triple Risk Model (*N* = 218)

Triple Risk Model (TRM)	Scopes	Factors
TRM 1: Vulnerability of the infant (39)	Abnormality, dysfunctionality (6)	Brainstem problems (28); arousal during sleep (23); congenital malformation (9); autoresuscitation (4); crying/colic problem (3); milk allergy (3)
	Birth problems (4)	Birth type (5); presentation at birth (1); PROM (1); time at birth (1)
	Genetic factors (3)	Genetics (21); previous still-birth/interruption/infant death (3); family history of SIDS (2)
	General factors (11)	Male sex (67); short gestation (53); ethnicity (race) (39); postnatal complication (NICU admission, assisted ventilation, any neonatal problem) (16); Apgar score (6); fetal growth retardation (5); growth (growth index, weight on death) (3); feeding problem (2); history of resuscitation (2); birth interval (2); undernutrition (1)
	Prenatal environment (15)	Prenatal smoking (65); prenatal care (14); multiple gestation (13); prenatal alcohol (11); GDM/PIH (6); prenatal illicit drug use (6); maternal infection (5); parental psychiatric illness (4); fetal hypoxia (3); smoking before pregnancy (2); maternal BMI (1); chronic maternal disease (1); hb, hct during pregnancy (1); unplanned pregnancy (1); weight gain during pregnancy (1)
TRM 2: Exogenous stressors (44)	Ecological factors (3)	Outside temperature (season, ambient/cold temperature) (32); weekday (4); air pollution (1)
	Circumstantial factors: feeding, ; sleeping; surroundings (22)	Sleep position (96); bed-sharing (48); environmental smoking (46); type of feeding (37); rooming-in (36); event place (car seat, sofa, couch, *ondol*, swing, etc) (30); head covering (feet-to-foot, sleeping bag, tight beddings, duvet) (27); overheating (wearing a hat, tog, the thermal overall grade of a covering/dress, extra warming, room temperature, warm type bedding, more blanket at night) (26); soft object near bedding (pillow, toy) (26); soft surface (mattress, soft bedding, sheepskin, waterproof bedding) (23); pacifier (22); parental alcohol use after birth (17); parental illicit drug use after birth (11); face position (9); event location (home, daycare, hotel, etc.) (8); sweating (7); swaddling (6); last feeding (5); beddings standards (2); moving (1); plastic covered mattress (1); paternal alcohol use (1)
	Clinical factors (13)	Infection (24); apnea episodes (16); vaccination (11); poor conditions (illness, intake reduction, sleeping more) (10); clinic visit (6); ALTE history (5); GER (4); antibiotics (3); age at weaning (2); complex chronic condition (2); drowsy when awake (2); eczema (1); fever (1)
	Socioeconomic status (6)	Parental age (43); parental education (31); gravidity/parity (31); marital status (28); economy (medical insurance, car owned, mortgage, overcrowding, poverty, underserved region, social welfare) (25); employment/job (17)
TRM 3: Critical period	Critical period (1)	Critical period (69)

SUDI=Sudden unexpected death in infancy; PROM=Prelabor rupture of membranes; SIDS=Sudden infant death syndrome; NICU=Neonatal intensive care unit; GDM=Gestational diabetes mellitus; PIH=Pregnancy induced hypertension; BMI=Body mass index; ALTE=Apparent life-threatening event in infancy; GER=Gastroesophageal reflux.

### Publication trends of SUDI articles

**Fig. 2:**
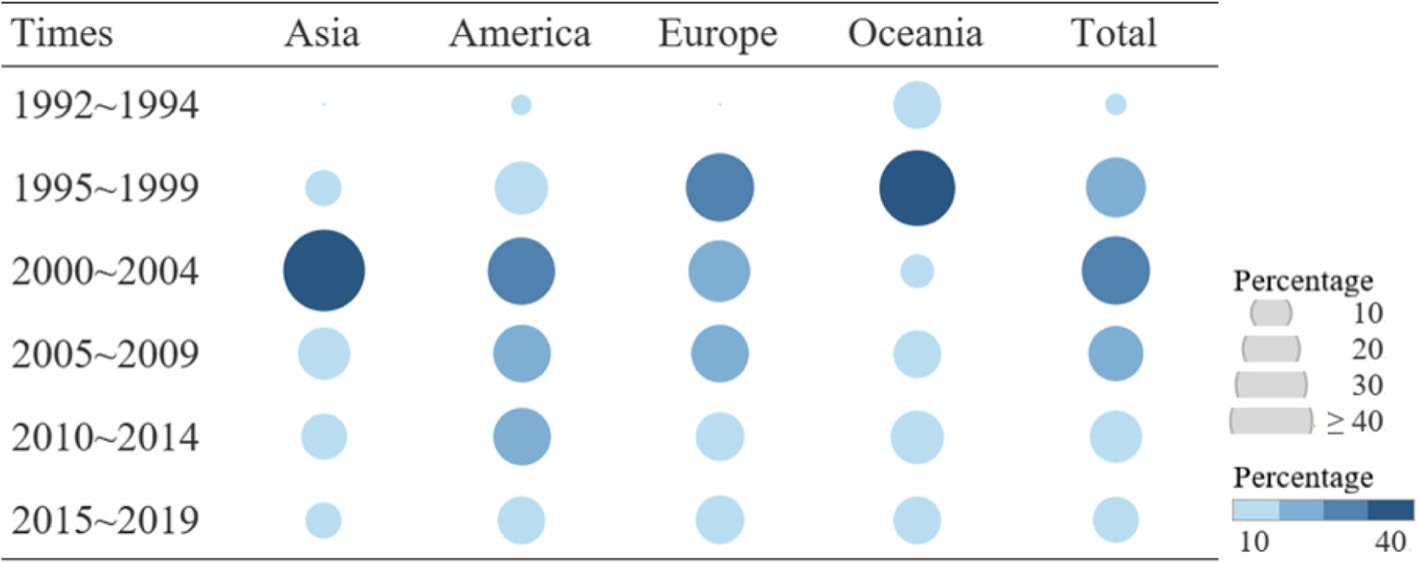
Proportional comparison of SUDI publication trends across regions by time

The 218 articles were from 26 countries divided into four geospatial cultural regions: Asia, America, Europe, and Oceania (Table 3). Most articles were published in America (81) or Europe (70). The US accounted for the most articles (73) followed by the UK (23), Japan (22), Australia (18), and New Zealand (10). Most of the Asian articles were reported from Japan (22) and South Korea (hereafter, Korea) (13). When the time of publication was categorized by 5-year intervals (with the exceptions of 3 years for 1992–1994 and 4 years and 4 months for 2015 to April 2019), the overall number of articles rapidly increased in 1995–1999 and 2000–2004, and then decreased gradually. This pattern was apparent in Asia, America, and Europe, where only two articles were initially identified. However, Oceania revealed a distinctive pattern, with a substantial amount of publications during the 1990s, but few articles in 2000–2004, when most of the articles were published worldwide. Fig. 2 presents the proportional trends in the publication of SUDI-related articles in these four regions by time.

**Table 3:** Frequency of SUDI Publications by Regions across Time

Times^†^	Regions^*^				Total (N = 218)
	Asia (*n* = 38)	America (*n* = 81)	Europe (*n* = 70)	Oceania (*n* = 29)	
1992 ~ 1994	0 (0.0)	2 (2.5, 33,3)	0 (0.0)	4 (13.8, 66.7)	6 (2.8, 100.0)
1995 ~ 1999	3 (7.9, 6.4)	14 (17.3,29.8)	20 (28.6, 42.6)	10 (34.5, 21.3)	47 (21.6, 100.0)
2000 ~ 2004	21 (55.3, 34,4)	22 (27.2, 36.1)	16 (22.9, 26.2)	2 (6.9, 3.3)	61 (28.0, 100.0)
2005 ~ 2009	6 (15.8,15.0)	16 (19.8, 40.0)	14 (20.0, 35.0)	4 (13.8, 10.0)	40 (18.3, 100.0)
2010 ~ 2014	5 (13.2,13.9)	16 (19.8, 44.4)	10 (14.3, 26.3)	5 (17.2, 13.9)	38 (16.5, 100.0)
2015 ~ 2019	3 (7.9, 10.7)	11 (13.6, 39.3)	10 (14.3, 35.7)	4 (13.8, 14.3)	28 (12.8, 100.0)
Total	38 (100.0, 17.4)	81 (100.0, 37.2)	70 (100.0, 32.1)	29 (100.0, 13.3)	218 (100.0, 100.0)

^*^ Asia: Japan 22; Korea 13; China, Hong Kong, Iran 1, *America: United States 73; Canada 7; Brazil 1, * Europe: United Kingdom 23; Germany, Norway 8; Sweden 5; France, Ireland 4, Austria, Italy 3; Netherlands, Spain 2; Belgium, Denmark, Lithuania, Romania 1, European Union multi countries 5, * Oceania: Australia 18; New Zealand 10; Indonesia 1

^†^ 3 years for 1992–1994; 4 years and 4 months for 2015–2019

*SUDI* Sudden unexpected death in infancy

### SUDI factors by regions and cultures

We explored how the top-ranked SIDS factors for each TRM category varied across regions (Table 4). For TRM 1 (vulnerability of the infant), the most frequent factor was brainstem problems in Asia, male sex and ethnicity in America, prenatal smoking in Europe, and male sex in Oceania. For TRM 2 (exogenous stressors), sleep position was most frequently mentioned in all regions, followed by bed-sharing in Asia and America, environmental smoking in Europe, and feeding type in Oceania. Sixty-nine (31.7%) of the 218 articles mentioned the critical period of age as a SUDI-related factor; this factor was most commonly mentioned in European articles (33) and the least frequently by articles from Oceania (2). The average number of factors addressed per article were 2.0 (2.3) for TRM 1, 3.4 (4.1) for TRM 2, and 0.3 (0.5) for TRM 3. The number of factors per article showed significant difference among the four regions for TRM 1 (F = 2.661, *p* = .049), TRM 2 (F = 3.537, *p* = .016), and TRM 3 (F = 5.892, *p* = .001). Overall, a total of 5.7 (5.7) factors were addressed per article, and European articles mentioned the most factors, at 7.7 (7.3) per article.

**Table 4:** Top 5 SUDI Factors by Regions (N = 218)

Factors		Regions				Total (N=218)
		Asia (n=38)	America (n=81)	Europe (n=70)	Oceania (n=29)	
TRM 1: Vulnerability of infant	Male sex	7 (18.4)	28 (34.6)	25 (35.7)	7 (24.1)	67 (30.7)
	Prenatal smoking	2 (5.3)	23 (28.4)	34 (48.6)	6 (20.7)	65 (29.8)
	Short gestation	3 (7.9)	18 (22.2)	26 (37.1)	6 (20.7)	53 (24.3)
	Ethnicity	1 (2.6)	28 (34.6)	6 (8.6)	4 (13.8)	39 (17.9)
	Brainstem problems	15 (39.5)	7 (8.6)	5 (7.1)	1 (3.4)	28 (12.9)
	M (SD) per article (0~39)	1.6 (1.2)	2.0 (2.6)	2.5 (2.4)	1.3 (1.6)	F = 2.661 (*p* = .049)
		2.0 (2.3)				
TRM 2: Exogenous stressors	Sleep position	14 (36.8)	39 (48.1)	30 (42.9)	13 (44.8)	96 (44.0)
	Bed-sharing	5 (13.2)	24 (29.6)	13 (18.6)	6 (20.7)	48 (22.0)
	Environmental smoking	3 (7.9)	14 (17.3)	22 (31.4)	7 (24.1)	46 (21.1)
	Parental age	3 (7.9)	16 (19.8)	19 (27.1)	5 (17.2)	43 (19.7)
	Feeding type	1 (2.6)	8 (9.9)	20 (28.6)	8 (27.6)	37 (17.0)
	M (SD) per article (0~44)	2.5 (3.3)	3.0 (3.2)	4.7 (5.3)	2.8 (3.4)	F = 3.537 (*p* =.016)
		3.4 (4.1)				
TRM 3: Critical period	Mentioned	10 (26.3)	24 (29.6)	33 (47.1)	2 (6.9)	69 (31.7)
	M (SD) per article (0~1)	0.3 (0.4)	0.3 (0.5)	0.5 (0.5)	0.1 (0.3)	F = 5.892 (*p* =.001)
		0.3 (0.5)				
M (SD) of all factors per article (0~84)		4.3 (3.8)	5.3 (4.8)	7.7 (7.3)	4.2 (4.5)	F = 4.653 (*p* =.004)
		5.7 (5.7)				

SUDI=Sudden unexpected death in infancy

**Fig. 3:**
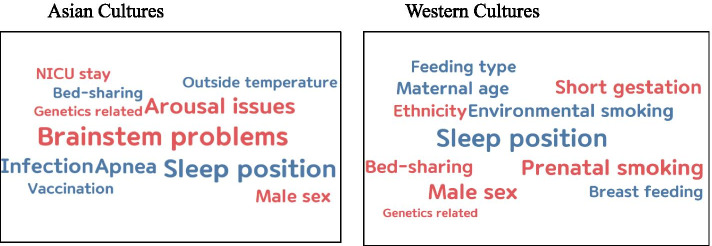
Comparison of the most commonly identified SUDI-related factors between Asian and Western cultures (vulnerability of the infant in red; exogenous stressors in blue)

Allocation of the articles into Asian and Western geo-cultures (corresponding to America, Europe, and Oceania) in the context of sociocultural structures resulted in 38 articles from Asian cultures, and 180 articles from Western cultures. We then identified the most frequently mentioned SUDI factors in articles from these two cultural zones. Table 5 and Fig. 3 show the most commonly mentioned factors of TRM 1 and TRM 2, with the exclusion of TRM 3 because of its more definitive nature (as a chronological factor) and the fact that it was mentioned relatively infrequently. Four common factors were identified in both cultural zones: sleep position, male sex, bed-sharing, and genetics. However, seven (63.6%) of the top 11 factors were independent in each cultural zone. More specifically, brainstem problems, arousal issues, apnea, and infection were among the top 5 factors in Asian articles, but did not belong to the top 11 factors of the Western articles. In the Western articles, prenatal smoking, short gestation, and environmental smoking were in the top 5, but were not found among the top 11 factors of Asian articles. Fig. 3 presents a visual comparison of variations in the most commonly identified factors for TRM 1 (red) and TRM 2 (blue) between Asian and Western articles.

**Table 5:** Top-ranked SUID Factors between Asian and Western Geo-cultures

TRM	Asian cultures (n = 38)			Western cultures (*n* = 180)		
	Rank	Factors	n (%)	Rank	Factors	n (%)
TRM1: Vulnerability of infant	1	Brainstem problems	15 (39.5)	1	Prenatal smoking	63 (35.0)
	2	Arousal issues	11 (28.9)	2	Male sex	60 (33.3)
	3	Male sex	7 (18.4)	3	Short gestation	50 (27.8)
	4	NICU stay	5 (13.2)	4	Ethnicity	38 (21.1)
	5	Genetics	4 (10.5)	5	Genetics	17 (9.4)
TRM 2: Exogenous stressors	1	Sleep position	14 (36.8)	1	Sleep position	82 (45.6)
	2	Apnea; infection	9 (23.7)	2	Environmental smoking; bed-sharing	43 (23.9)
	4	Bed-sharing; outside temperature; Vaccination	5 (13.2)	4	Maternal age	40 (22.2)
				5	Feeding type	36 (20.0)
				6	Breastfeeding	34 (18.9)

### Sleep practices related to SUDI between the two cultural zones

Since we sought to explore culture-specific sleep practices that may contribute to SUDI, we retrieved the 10 sleep-related factors from TRM 2 (exogenous stressors) and compared their frequency between the two cultural zones. As shown in Table 6, a total of 320 observations (147%) of 10 factors were identified in 218 articles: 37 (17%) in articles from Asian cultures and 283 (129.8%) in articles from Western cultures. Both cultural zones identified sleep position (44.0%), bed-sharing (22.0%), and rooming-in (16.5%) as the three most common sleep-specific factors for SUDI. Sleep position involves various types and patterns, such as supine and prone positions for the infant’s routine sleep or at the scene, or a history of position change in terms of gross motor development. Bed-sharing includes issues such as co-use or sharing of sleep materials (i.e., the same surface, mattress, and/or sheets) during sleep. Rooming-in occurs when multiple persons, including the infant, sleep together in the same room or space, without the infant sharing sleep materials with any other person(s).

**Table 6:** Comparison of Sleep-related Factors in Articles from Asian and Western Cultures (N = 218)

Sleep-related factors	Observation	Contents	Asian cultures (n = 38)	Western cultures (n = 180)	χ^2^ (*p*)
Sleep position	96 (44.0)	Lateral, prone, supine position for routine or at the scene, or history of position change	14 (36.8)	82 (46.6)	0.967 (.371)
Bed-sharing	48 (22.0)	Sharing bed, mattress, pad, sleep surface, etc.	5 (13.2)	43 (23.9)	2.104 (.196)
Rooming-in	36 (16.5)	Sleeping in the same room or space	4 (10.5)	32 (17.8)	1.197 (.342)
Place of death	30 (13.8)	Car seat, couch, *ondol*, sofa, swing	3 (7.9)	27 (15.0)	1.335 (.309)
Head covering	27 (12.4)	Unfixed sheet over face, sleeping bag or heavy duvet to cover face, feet to foot for preventive effect, etc.	4 (10.5)	23 (12.8)	0.147 (1.000)
Overheating	26 (11.9)	Temperature at the time of the incident; wearing a hat, the tog of a sheet or cloth, extra heating device, room temperature, extra sheet	1 (2.6)	25 (13.9)	3.785 (.055)
Soft objects near bedding	26 (11.9)	Pillow, toy	3 (7.9)	23 (12.8)	0.712 (.583)
Soft or non-breathing surface	23 (10.8)	Mattress, soft bedding, sheepskin, waterproof mattress or sheet	3 (7.9)	20 (11.1)	0.344 (.773)
Swaddling	6 (2.8)	Wrapping to restrict arm movements using sheets, cloth or goods	0 (0.0)	6 (3.3)	1.303 (.593)
Bedding standards	2 (0.9)	Accreditation or regulation on bed safety of cribs, mattresses, frames, etc.	0 (0.0)	2 (1.1)	0.426 (1.000)
Total	320 (147%)		37 (17.0)	283 (129.8)	

* Fisher’s exact test

**Fig. 4:**
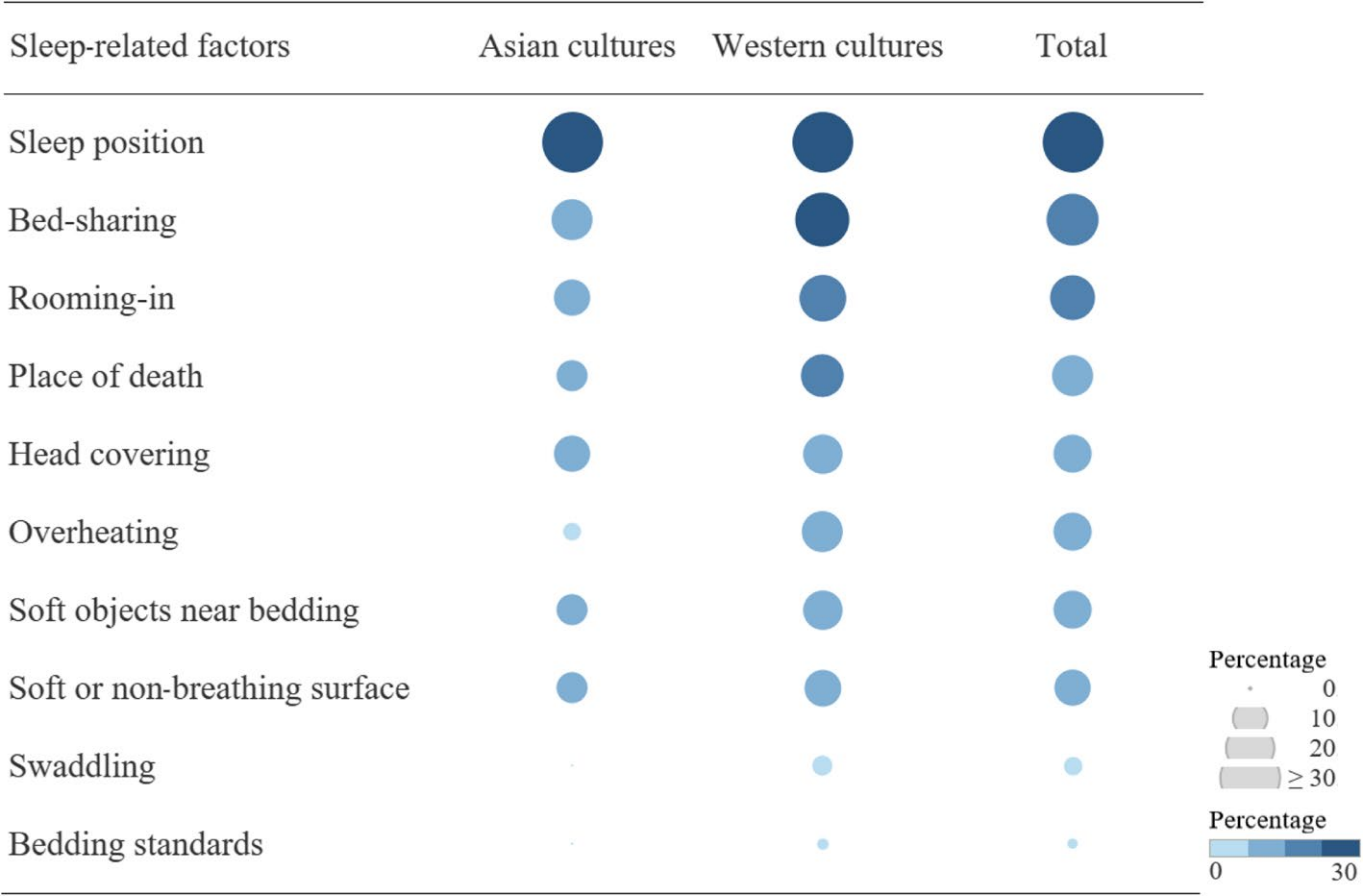
Proportional comparison of sleep-related SUDI factors identified in articles from Asian and Western cultures

Regarding locations associated with SUDI, Western articles reported places such as car seats, couches, and swings, whereas Asian articles described underground heated floor surfaces (known as *ondol* floors in Korea). Articles from both cultural zones similarly reported various patterns of head covering as a risk factor for SUDI, such as unfixed sheets, sleeping bags, or the use of a heavy duvet, while the “feet-to-foot” practice played a protective role. Overheating issues included wearing a hat, sheets, or supplements for extra warming; hot room temperatures; and thermal overall grade of a covering (TOG). Surprisingly, only 1 out of the 38 Asian articles (2.6%) reported overheating as a SUDI-related factor, while 25 out of 180 Western articles (13.9%) did so. Soft objects near bedding and soft bedding surfaces were also addressed as SUDI risks in articles from both cultural zones. However Asian articles did not mention either swaddling or bedding standards as SUDI-related factors, while Western articles did so. In fact, Western articles addressed each of the 10 sleep-related factors more frequently than Asian articles, as shown in Fig. 4.

## Discussion

Recent research and statistics on SUDI rates suggest that some SUDI-related factors need to be understood in cultural contexts when they occur during sleep. In this review article, we present a generalized overview of the SUDI factors reported worldwide as classified into TRMs, along with an analysis of the distribution of those factors across geo-cultural regions over time. The quantity of SUDI-related articles sharply increased several years after the first AAP position paper on SIDS. Not every country in the world reports SUDI rates, and in our analysis of related factors, we identified only 26 countries for 218 articles, corresponding to slightly more than 10% of all countries in the world. Nevertheless, we identified several sociocultural issues including sleep practices that might have significant implications for understanding yet-to-be-revealed mechanisms of SUDI-related factors.

First, the quantity of SUDI articles was imbalanced across geo-cultural regions, which may impede a comprehensive understanding of cultural perspectives on SUDI-related factors. Our findings showed that 17.4% of articles were from Asia, whereas 69.3% were from America and Europe. In accord, most SUDI statistics and research are from Australia, the UK, the US, or Scandinavian countries [18]. However, a lower quantity of SUDI-related articles does not indicate a lower incidence of SUDI in a given society, or vice-versa. In affluent societies with high-quality welfare systems, clear evidence has been documented of low SIDS rates such as 0.09 per 1000 live births in Denmark, 0.14 in Sweden, and 0.16 in Norway [19]. Contrarily, if accurate diagnoses may not be readily feasible and the resources for infant mortality rate or SUDI statistics remain limited in some developing societies, low reported rates may not reflect an actually low incidence, and the extent of SUDI or SIDS as a problem may be still uncertain in some Asian societies.

Recent reports from multiethnic societies such as England and the US have underscored the need to explore sleep practices of ICP in Asian societies with low SUDI rates in order to develop SIDS-reducing strategies [2, 14]. In Korea, there has been an urgent demand for accurate estimations of SUDI rates and comprehensive research on SUDI for decades. Since 2016, Korea has had a universal nationwide registry system for all births and infant deaths, and legally obligatory autopsies are conducted for all unexplained deaths or any unnatural deaths without a diagnosed cause or manner of death [20]. A recent article from Japan described a similar process of standardizing a protocol to report SUDI as the first extensive epidemiologic survey [21]. The establishment of a SUDI investigation and reporting protocol as launched in Korea or Japan would stimulate comprehensive research to broaden cultural perspectives on SUDI-related factors, with an emphasis on the effects of sleep practices in Asian cultures.

Secondly, careful consideration is required in understanding the cultural mechanisms of sleep-related SUDI factors across cultures. Bed-sharing and rooming-in, which were substantially identified in both two cultural zones, are significant exemplars of this point. In our study, Western and Asian articles identified bed-sharing and rooming-in as the most commonly reported sleep-specific factors, along with sleep position. However, an in-depth exploration suggests that there could be a substantial difference in these phenomena between cultures. Bed-sharing, also called ‘co-sleeping,’ is defined as the infant sleeping on the same surface with another person [22]. In Western culture, an independent bed is common, as infants tend to sleep in their own crib or cot. The surface of the bed is clearly separated from the floor of the room, which may be considered as an unclean zone to sleep, as the room floor may be open to outdoor shoes. Therefore, the bed is the only place for people to sleep; under such circumstances, bed-sharing may serve as a SIDS risk factor by increasing the possibility of hypoxia, suffocation, or overheating, particularly in crowded settings when an infant is placed between adult sleepers on a soft mattress.

However, in Asian countries such as Korea, sleeping on the floor is a traditional practice, and people prefer hard mattresses that resemble a wood floor (and sometimes even use stone mattresses). Any floor surface is expected to be clean and easily ready for family members to sit and lay down upon, as one does not wear outdoor shoes inside the home. Furthermore, the bed mattress is not high, and sometimes only a thin mattress (like a topper) is used without a bedframe to enjoy the warm surface directly, as in the *ondol* system. These conditions make it more likely for infants and their primary caregiver, mostly the mother, to sleep together on the floor of a room and even to promote breastfeeding, while the father and other family members sleep on the bed or in separate rooms to prepare for the next workday. Furthermore, traditional Chinese medicine supports the benefits of a warm floor surface for postpartum recovery and extrauterine adaptation of infants based on the theory of *yin* and *yang* balance [23]. In Asian cultures, often the sleeping surface for an infant is not the surface of a separate bed, but the floor of the room, meaning that bed-sharing is considered to be analogous to rooming-in, which is considered to be preventive against SUDI. Although bed-sharing involves shared bedding to some extent, the method of bed-sharing in Asia is quite different from that in Western countries, where bed-sharing means bringing the baby into bed with both parents [24].

More variations in sleep-related SUDI factors were observed between the two cultural zones, as shown in Table 6; for instance, swaddling or bedding standards were not reported in Asian articles at all. Overheating was mentioned only once in Asian articles. This implies that some sleep-related SUDI factors may not be articulated or conceptualized in a way that is sufficiently sensitive to cultural circumstances.

Sleep customs (i.e., where and how to sleep, and with whom) are evolutionary practices; therefore, the meaning and application of specific sleep practices may differ across cultures. This underscores the need to clarify the specific definition of bed-sharing and rooming-in from the perspective of ICP and house living structure. More scrutiny is also needed regarding the relationship between other sleeping practices and SUDI, as the effect size of soft mattresses and bed-sharing could be still very substantial in Western cultures, or the effect size of overheating could be underestimated in Asian cultures that tend to favor warmth of the floor surface.

Third, a striking finding in our study relates to cultural variations in identifying smoking as a SUDI factor. Western articles placed a strong emphasis on the negative effects of any type of smoking on SUDI; smoking was categorized into two separate factors (prenatal smoking and environmental smoking) as top-ranked addressed factors. However, Asian articles paid little attention to this issue, as only 2 articles dealt with prenatal smoking and 3 articles pointed out environmental smoking. Along with evidence from throughout the world supporting the beneficial effects of smoking cessation on infant health promotion [25, 26], Western countries continue to emphasize smoking prevention and cessation, as the Collaborative Improvement and Innovation Network (CoIIN) of the National Institute for Children’s Health Quality (NICHQ) announced SIDS/SUDI safe sleep practices and smoking cessation as the top-priority strategy to reduce infant mortality [27].

In contrast, in traditional Asian cultures, smoking has been viewed permissively as a matter of manhood [28]. Regardless of improvements in women’s social status and economic independence, the female smoking rate may be underemphasized due to persistent social stigma against female smoking in many Asian countries [28, 29]. Since the mother is the most-likely primary caregiver during early infancy, she could be socially discouraged from smoking, and smoking history may be less frequently disclosed in SUDI cases in Asia [30]. This may explain the discrepancies in smoking as a SUDI risk between two cultural zones. Hidden smoking involves multiple jeopardy through social isolation surrounding smoking and the lack of a chance for a smoking cessation intervention, in addition to the negative effects of smoking itself [31]. This underscores the vital need to unveil the degree of female hidden smoking in Asian countries and to explore its effects on SUDI.

Besides these findings, our study showed that approximately two-thirds of the top-ranked SUDI factors were independent between the two geo-cultural zones. Due to the uniqueness of each factor to SUDI and the limited numbers for each factor in Asian articles, it might be premature to make an inferential conclusion regarding these differences. Nonetheless, these findings clearly illustrate the possibility of cultural variations in SUDI-related factors, including both sleep practices and vulnerable characteristics of infants, as well as other exogenous factors.

Our study has several limitations hindering the generalization of the findings for universal applications. First, we applied neither the San Diego definition of SIDS nor the wider SUDI classification for database search. Rather we used specific search terms after 1992 to focus on the identification of relatively well-confirmed sleep-related SUDI factors (from the thousands of putative SUDI-related factors) to produce a relatively explicit and narrow search pool of 441 articles. This resulted in the exclusion of the SUDI focused studies on safer sleep practices and several of the case-control and cohort studies that initially identified many of the main sleep-related risk factors. Therefore, our search conditions may not have been fully suitable for retrieving a relatively neutral, fully comprehensive range of factors and circumstances associated with sleep-related infant death. Second, the cultural zone of each article was determined based on the country of the study, rather than the ethnicity of the study subjects. Therefore, it is possible that articles with subjects from multiple ethnic groups may have naturally covered various cultural aspects of SUDI, but were classified as belonging to a single cultural zone in this study. Third, the decision to allocate the counties of the articles into four regions and two cultural zones was performed on a purely geographic basis. Therefore, the link may have been subjective in some countries; for instance, Brazil was classified as an American country, but may not have a prototypically Western culture.

These limitations, along with the substantial findings of the study, suggest the need for future research on cross-cultural differences in SUDI in several directions, including a comparison of the effect sizes of sleep-related SUDI factors among various cultures, or identification of benign/preventive factors against SUDI from societies with low SUDI rates. Furthermore, a systematic review using a computerized algorithm with wide-ranging search conditions would increase the depth and scope of knowledge on SUDI. As a general implication of these findings, child health professionals need to develop SUDI reduction strategies with professional awareness and collective knowledge on sociocultural variations related to the circumstances of SUDI in a given society.

## Conclusion

In this study, we explored variations in SUDI-related factors from 218 articles with an emphasis on sleep practices across time and two geo-cultural regions (Western and Asian countries). Despite the urgent need to explore ICP and the caregiving environment in low-SUDI societies, Asian cultures showed a significant lack of articles on SUDI. Several sociocultural issues on sleep practices were recognized, such as the meaning of bed-sharing and rooming-in, residential styles, and traditional health beliefs; these cross-cultural differences should be kept in mind when evaluating SUDI-related factors. The fact that Asian articles paid little attention to smoking was striking, whereas Western articles placed a strong emphasis on smoking as a SUDI risk factor. Collectively, these findings suggest a need to enhance SUDI reduction strategies by incorporating gender-sensitive smoking cessation interventions. Child health professionals must be alert to sociocultural variations in sleep practices and SUDI-related factors, and should act preemptively to promote SUDI reduction strategies. Research on the underlying mechanisms that regulate sleep-related SUDI should continue with an even more in-depth focus on sociocultural circumstances.

## Data Availability

The datasets used and/or analysed during the current study are available from the corresponding author on reasonable request.

## Abbreviations

SUDI: Sudden Unexpected Death in Infancy
SIDS: Sudden Infant Death Syndrome
TRM: Triple Risk Model
